# Contralateral Neck Irradiation Can Be Omitted for Selected Lateralized Oral Cancer in Locally Advanced Stage

**DOI:** 10.3390/curroncol29100547

**Published:** 2022-09-26

**Authors:** Yung-Jen Cheng, Hsin-Ying Lin, Mu-Hung Tsai, Tzu-Hui Pao, Chia-Hsiang Hsu, Yuan-Hua Wu

**Affiliations:** 1 Department of Radiation Oncology, National Cheng Kung University Hospital, College of Medicine, National Cheng Kung University, Tainan 704302, Taiwan; 2 Department of Ophthalmology, Chi Mei Medical Center, Tainan 71004, Taiwan; 3 Institute of Computer Science and Information Engineering, National Cheng Kung University, Tainan 704302, Taiwan; 4 Department of Emergency Medicine, National Cheng Kung University Hospital, College of Medicine, National Cheng Kung University, Tainan 704302, Taiwan

**Keywords:** oral cancer, adjuvant radiotherapy, contralateral irradiation, contralateral neck failure, survival analysis

## Abstract

(1) Background: To investigate the contralateral neck failure (cRF) rates and outcomes among patients with well-lateralized locally advanced oral cavity squamous cell carcinoma (OSCC) with/without ipsilateral or bilateral neck adjuvant irradiation. (2) Methods: Patients with lateralized OSCC diagnosed between 2007 and 2017 were retrospectively enrolled. Patients who had undergone curative surgery with pathologically proven pT3/4 or pN0-2b without distant metastasis were included, while those with cross-midline, neck-level 1a involvement and positive extra-nodal extension (ENE) were excluded. The primary endpoint was the cumulative incidence of 5-year cRF as the first site of failure. The secondary endpoints included cancer-specific survival (CSS), local-regional recurrence-free survival (LRRFS), distant-metastasis-free survival (DMFS), and contralateral-regional recurrence-free survival (cRRFS). (3) Results: In total, 149 patients were analyzed with a median follow-up time of 5.2 years (range, 2.91–7.83). Pathological stages T3 and T4 were 22.7% and 56.8%, respectively. Pathologically negative and positive lymph nodes were 61.4% and 38.6%, respectively. The cumulative 5-year cRF rate was 3.6% (95% CI, 1.3–7.7%). No significant differences in the 5-year CSS, LRRFS, DMFS, and cRRFS were observed among those undergoing unilateral or bilateral neck irradiation. Five patients (3.4%) had contralateral neck recurrence, all simultaneously with local recurrence. No isolated contralateral neck recurrence was identified. (4) Conclusions: The cRF rate was acceptably low in patients with well-lateralized advanced OSCC with the initially uninvolved contralateral neck. Omitting contralateral neck irradiation with active surveillance could be considered without compromising the cure rate in locally advanced OSCC patients.

## 1. Introduction

In patients with oral cavity squamous cell carcinoma (OSCC), neck metastasis is an important prognostic parameter to determine the treatment outcome [1]. It is generally accepted that elective neck treatment, either dissection or radiotherapy (RT), is the usual choice for the clinically N0 neck with ≥20% probability of occult neck metastasis [2]. However, the decision to administer elective treatment to the contralateral neck, which has a low recurrence risk, depends on the discretion of the physician.

Historically, contralateral neck failure rates of 0.9–34.7% have been reported from oral carcinoma [3,4]. This wide range of rates may be attributed to various factors, including tumor extension, tumor status (e.g., T- or N-stage, histologic grade), number of lymph nodes involved, or the presence of extranodal extension (ENE) [5,6,7]. Some analyses in the literature have reported a higher contralateral neck metastasis rate with a positive ENE status and cross-midline primary OSCC [8,9], while some studies have reported a low rate of cRF (<6%) in patients with well-lateralized resected oropharyngeal/oral cavity cancer [5,10,11,12,13,14]. Another recently published prospective phase II study (*n* = 72) demonstrated a good control rate (97%; 95% CI: 93.4–100%) in unirradiated neck in pathologically node-negative head and neck cancer [15]. However, most of these studies contained groups with relatively early-stage disease (T1-2) and/or included cancer sites with mixed entities of head and neck cancer. In addition, no current randomized control trial could present any robust evidence for recommending contralateral neck-sparing irradiation. As a result, no standard practice has yet been developed for managing contralateral nodal-negative OSCC, especially in lateralized advanced stages.

In the past, once radiotherapy was administered, bilateral neck lymph nodes would be electively irradiated in most of the patients with contralateral nodal-negative OSCC. This treatment paradigm was empirically based on series from an old era [16,17], and nodal staging evaluation in those series was also exclusively based on clinical examination instead of advanced diagnostic techniques such as neck ultrasound, computed tomography (CT), magnetic resonance imaging (MRI), positron emission tomography scan (PET-CT), or sentinel node biopsy. More recently, these modern techniques have significantly improved the accuracy and reliability of nodal staging, reducing the possibility of occult nodal metastasis and avoiding unnecessary treatment.

This study investigates the cRF rate and outcomes in a group of patients with well-lateralized locally advanced OSCC with/without ipsilateral or bilateral neck irradiation. The study aims to corroborate the result obtained after omitting the contralateral neck irradiation field without compromising the cure rate in such OSCC patients.

## 2. Materials and Methods

### 2.1. Selection of Patients

Patients diagnosed between 2007 and 2017 with oral cavity cancer were identified. The inclusion criteria were as follows: patients diagnosed with lateralized oral cancer (buccal and cheek, gum, and retromolar subsites), curative surgery with pathologically proven pT3/4 or pN0-2b, and no metastasis before treatment. The exclusion criteria were as follows: nonlateralized-tendency oral cancer (oral tongue and floor of the mouth subsites) or unknown subsite, previous cancer or RT history before the diagnosis of oral cancer, simultaneously diagnosed other cancers, nonsquamous cell histology type, pathological proven neck-level 1a involvement, or bilateral neck lymph node involvement (stage N2c). Pathologically proven ENE of the positive lymph node was also excluded because of the high tendency of treating prophylactic contralateral neck fields by radiation oncologists. Patients with an obvious central disease or in whom the initial tumor invaded beyond the midline or was within 1 cm from the midline were also excluded. All patients had undergone the pretreatment workup that included chest X-ray, abdominal sonography, bone scan, and baseline laboratory blood test. Head and neck images (either CT or MRI at the minimum) had to be obtained before beginning the treatment. The use of PET-CT, chest CT, and neck sonogram was optional and depended on the clinician’s discretion. Tumor staging was conducted based on the American Joint Committee on Cancer (AJCC) staging system 6th and 7th Edition [18,19]; the two editions did not report any significant changes for the oral cavity cancer. This study was reviewed and approved by the Institutional Review Board (IRB) of our institute (National Cheng Kung University Hospital [IRB number: A-ER-111-181]) and conducted according to the ethical guidelines of the Declaration of Helsinki. The requirement for informed consent from the study subjects was waived due to the retrospective study design.

### 2.2. Treatments

Maximal curative resection to the primary tumor with uni- or bilateral neck dissection was conducted depending on the surgeon’s discretion. Surgical margins of >3 mm, 1–3 mm, and <1 mm were defined as free margin, close margin, and inadequate margin, respectively. The decision to undergo adjuvant radiotherapy was made based on the National Comprehensive Cancer Network (NCCN) guidelines. Patients with minor risks for recurrence (such as advanced T- or N-stage, perineural invasion, and lymphovascular invasion) were suggested to receive adjuvant radiotherapy, while adjuvant concurrent chemoradiotherapy was usually administered to those with a major risk for recurrence (such as an inadequate margin or positive ENE).

Once adjuvant RT was planned, CT simulation with a thermoplastic immobilization mask was performed for contouring the patients by physicians. An intensity-modulated radiation therapy (IMRT) technique was used for tailoring the treatment volume to maximize the coverage conformally while sparing the organs at risk. The IMRT field encompassed the primary surgical tumor bed, either unilateral or bilateral neck lymphatics, and some fields of the lower anterior neck using three-dimensional conformal techniques depending on the preference of the radiation oncologist. RT was given in doses of 1.8–2 Gy five times per week. The treatment guidelines included a prophylactic dose of 45–54 Gy to the elective clinical target volume (CTV), with a boost dose of 59.4–66.6 Gy to the high-risk CTV. Cisplatin-based chemotherapy was usually administered in concurrent settings (either 3-weekly 100 mg/m^2^ cisplatin, weekly 35–40 mg/m^2^ cisplatin or combined with fluorouracil was acceptable at the discretion of medical oncologists).

After treatment, the patients were regularly followed up every 3–6 months for physical and image examination. If the tumor recurred or the patients developed a second primary malignancy, salvage therapy was usually performed.

### 2.3. Outcomes and Statistical Analysis

The primary endpoint was to assess the cumulative 5-year cRF rate as the first site of failure. All-time cRF of all patients was also assessed from after the first definitive treatment until the last follow-up. Other endpoints, including cancer-specific survival (CSS), local-regional recurrence free survival (LRRFS), distant-metastasis free survival (DMFS), and contralateral-regional recurrence free survival (cRRFS), were also analyzed for the cohort using the Kaplan–Meier method. To test statistically significant differences between the curves, the log-rank test was used. All survival times were calculated immediately from the date of curative surgery. CSS was determined until death from the oral cavity cancer (patients dying from other cancers or causes were censored at the time of death). LRRFS and DMFS were defined to develop either local or regional recurrence and distant metastasis, respectively (any events or second primary diseases or lost follow-ups were censored at the time). The cRRFS was defined as the development of contralateral neck recurrence including, but not limited to, simultaneous local or distant failure, occurring even after second primary disease (any events or lost follow-ups were censored at the time). Univariate analyses were performed with the log-rank test to detect significant clinicopathological predictors for the cRRFS outcome. The pattern of first-site failure was documented as the local, ipsilateral, or contralateral neck, distant metastasis, or any combination thereof. All statistical analyses were performed using R software (version 3.6.1; R Foundation for Statistical Computing, Vienna, Austria). Two-tailed *p* values of <0.05 were considered statistically significant.

## 3. Results

### 3.1. Patient Characteristics

The patient characteristics are summarized in Table 1. In total, 149 patients (men = 145 (97.3%), women = 4 (2.7%)) were analyzed from 2007 to 2017. The median follow-up time of all patients was 5.2 years (range, 2.91–7.83 years). The median diagnostic age was 52 years (range, 47–59 years). The most common subsite was buccal and cheek (*n* = 95, 63.8%), followed by gum (*n* = 46, 30.9%) and retromolar region (*n* = 8, 5.4%). The proportions of pathological stages T3 and T4 were 22.7% and 56.8%, respectively, while those of pathologically negative and positive lymph nodes were 61.4% and 38.6%, respectively. All patients received neck dissection on at least one side, 18 patients received it on bilaterally (12.1%), and 131 received it on one side (87.9%). There were 5 (3.4%) patients who received PET-CT scan during the pretreatment workup. Only 15 (10.0%) patients reported having never smoked, consumed alcohol, or chewed betel quid.

### 3.2. Characteristic Differences between Different Treatment Approaches

A group of 44 patients received adjuvant RT with unilateral neck irradiation (UNI) and 34 with bilateral neck irradiation (BNI), while 71 patients did not receive any adjuvant RT. The no-RT group had more pN0 and free margin status than those in the adjuvant RT group (*p* < 0.05). In the adjuvant RT setting, no significant differences between the UNI and BNI groups were observed except for in the median diagnostic age (56 years in UNI and 50 years in BNI, *p* = 0.018) (Table 1). In addition, 36 patients (46.2%) of the adjuvant setting population received concurrent chemotherapy, with most concurrent chemotherapy regimens being cisplatin and fluorouracil (52.8%), then cisplatin alone (30.6%).

### 3.3. Cumulative 5-Year cRF Rate and Survival Analysis

**Figure 1:**
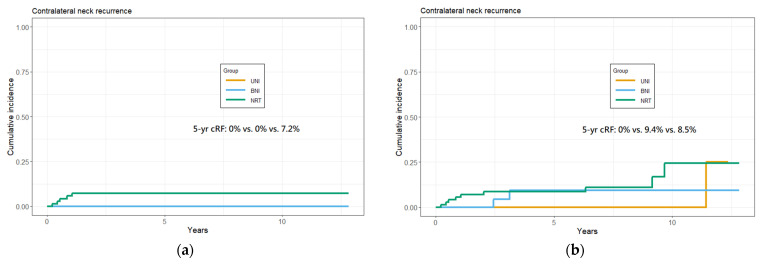
Cumulative 5-year cRF rate among patients in different groups. (**a**) Calculated with cRF as the first failure site. (**b**) Calculated with all-time cRF, which does not have to be the first failure site.

No cRF was identified as the first site of failure in both UNI and BNI groups, while it was reported in 7.2% of the no-RT group (Figure 1a). In the entire study cohort, the cumulative 5-year cRF rate was 3.6% (95% CI, 1.3–7.7%). Considering cRF at any time (not limited to first recurrence), the overall cRF rate after 5 years became 6.0% (95% CI, 2.8–10.9%) (Figure 1b).

**Figure 2:**
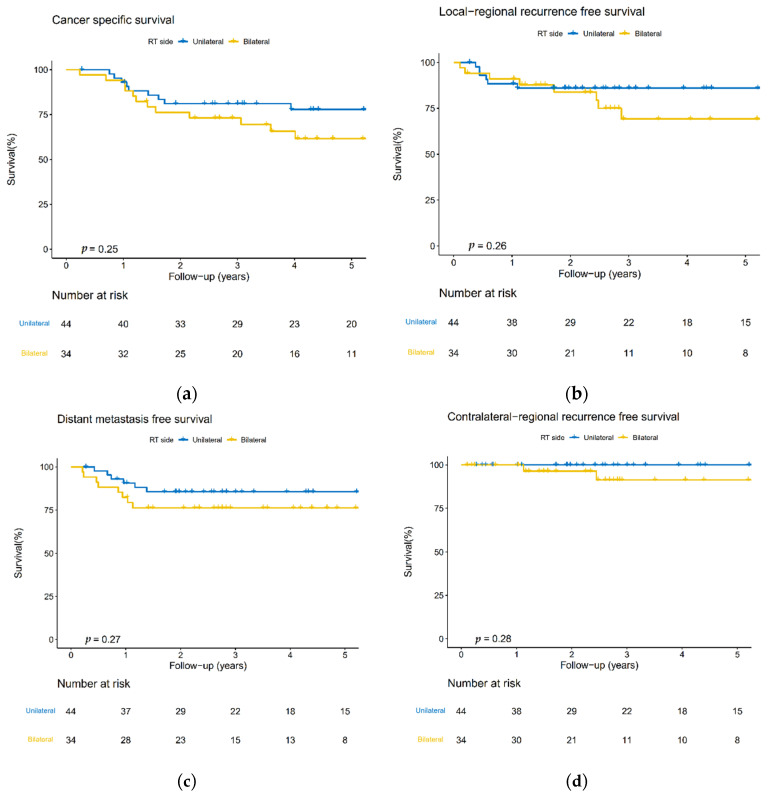
Kaplan–Meier plots of the 5-year (**a**) cancer-specific survival (CSS); (**b**) local-regional recurrence-free survival (LRRFS); (**c**) distant metastasis-free survival (DMFS), and (**d**) contralateral-regional recurrence-free survival (cRRFS) rates between the unilateral and bilateral neck irradiation groups.

No statistically significant differences were observed for 5-year CSS, LRRFS, DMFS, and cRRFS for the UNI and BNI groups (Figure 2). The 5-year CSS was 77.9% vs. 61.6% (*p* = 0.25); the 5-year LRRFS was 86.0% vs. 69.2% (*p* = 0.26); the 5-year DMFS was 85.6% vs. 76.3% (*p* = 0.27); and the 5-year cRRFS was 100% vs. 91.4% (*p* = 0.28). Univariate analysis did not detect any significant clinicopathological predictors for cRF (Table 2).

### 3.4. Disease-Failure Pattern

**Figure 3:**
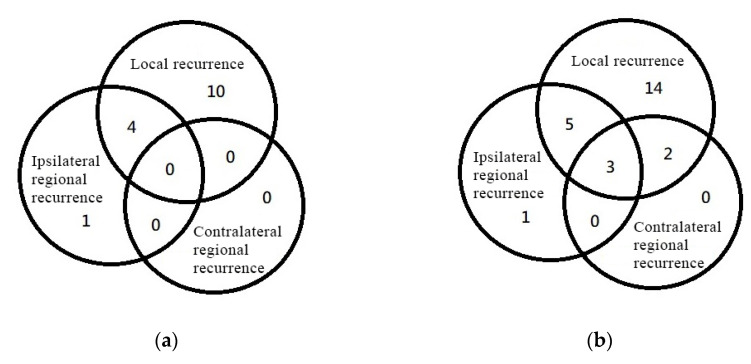
Locoregional failure patterns among (**a**) Fifteen patients with recurrent tumors in patients with neck irradiation; (**b**) Twenty-five patients with recurrent tumors in the entire cohort.

No contralateral neck recurrence was identified in the UNI and BNI groups (Figure 3a). Among the 15 patients (19.2%) who reported locoregional relapse, 2 were without any disease-free status, 9 had in-field recurrence (including the only one with isolated ipsilateral neck recurrence), 2 showed marginal recurrence (1 of them received delayed RT due to poor wound condition), and the remaining 2 patients showed distant metastasis before local recurrence.

No isolated contralateral neck recurrence was identified in the entire cohort. Five patients (3.4%) reported contralateral neck recurrence, all simultaneously with local recurrence (Figure 3b). Locoregional relapse was noted in 25 patients (16.8%), most with local failure only (56% of all relapsed patients). There was only one case of isolated ipsilateral neck recurrence (0.7%). In total, 12 patients (8.1%) had distant metastasis, 4 had combined local or regional relapse (2.7%), and 8 had distant metastasis only (5.4%).

## 4. Discussion

No consensus has yet been reached and no robust evidence is available on the benefits of contralateral neck irradiation for patients with contralateral nodal-negative OSCC. Furthermore, RT-associated acute and long-term toxicities are significantly impacted by the treatment volume in patients with head and neck cancer, especially those receiving trimodality therapy (radical surgery plus concurrent chemoradiotherapy). With improved radiation techniques, IMRT enables tailored treatment, maximizing target volume coverage while limiting the doses to normal tissues. As radiation oncologists aim to achieve a balance between the radiation toxicity and treatment outcome, the chance to omit the unnecessary treatment field without compromising the cure rate needs further investigation.

Recent retrospective data reported a low contralateral neck recurrence rate in patients with head and neck cancers. Table 3 lists a literature review of contralateral neck failure rate in primary head and neck cancers, particularly cancer at the oral cavity sites [5,11,12,13,14,15,20]. Unlike most of the historical analysis of mixed head and neck cancers and relatively early-stage OSCC, our study focused on a homogeneous patient series with well-lateralized and more advanced-stage OSCC to avoid confounding interactions between different origins of head and neck cancers. According to the results, this study demonstrates a comparably low cRF with that of patients with high-consistency advanced-stage OSCC.

In our cohort, the 5-yr cRF rate was as low as 3.6%. No cRF was identified in patients who received neck irradiation, either UNI or BNI. Hence, omitting the contralateral N0 neck might be a reasonable approach in such patients. This strategy was corroborated by previous studies. Vergeer et al. investigated 123 patients with oral cavity cancer (85%) and oropharyngeal cancer without contralateral neck irradiation and reported a cRF rate of 5.7% [5]; however, their group included 7% patients with close/cross midline disease, which could possibly increase the cRF. Wirtz et al. reported a cRF rate of 6.1%, mainly in the oropharynx (52.8%), with extended data for oral cavity (38%) and hypopharynx (10%) [12]; however, 73.1% of their patients received contralateral neck dissection, which could have been an overtreatment. Another phase II study demonstrated a low cRF rate of 2.8% for resected head and neck cancers. Similarly, 71% of patients had a cross-midline disease and up to 92% of patients received bilateral neck dissection before adjuvant RT [15]. Notably, our cohort, comprising 79.2% of patients with the T3/4 tumor, still revealed a reliably low contralateral neck recurrence rate, even though nearly 90% of them did not undergo contralateral neck dissection. Although contralateral neck dissection could be actively performed to detect occult contralateral nodal metastasis, there were certain short-term and long-term impacts on the quality of life of the patients [21].

Tumor-dependent factors that predicted contralateral neck recurrence are not well known and are still under investigation. The Sentinel European Node Trial included patients with lateralized, early T, and N0 tumors and demonstrated that the positive contralateral sentinel node was detected in only 1.9% of cases but in up to 6% of cases of midline tumor [22]. Al Mamgani et al. found midline involvement as the most significant correlation with cRF in a pooled analysis (12.12% with midline involved vs. 1.71% with free midline, *p* = 0.001) [23]. It is generally accepted that there is a high frequency of lymphatic vessels crossing the midline in certain tumor localizations (e.g., part of the oral cavity, tongue, and floor of the mouth) [24]. ENE is also regarded as a predictor of cRF. Two large retrospective studies identified ENE as a strongly independent risk factor for 5-yr cRF (HR: 12.978, 95% CI: 1.328–126.829, *p* = 0.028) and for cRF in patients showing local recurrence (HR: 4.957, 95% CI: 1.763–13.934, *p* = 0.002) [8,9]. As a result, one should be very cautious before deciding to omit contralateral neck irradiation in cases of midline crossing primary and ENE-positive status cancers. However, these patients were initially excluded from our study cohort because midline crossing primary and ENE-positive status cancers usually drive more extensive treatments (e.g., elective contralateral neck dissection and/or prophylactic contralateral neck field irradiation) by clinical physicians. A prompt discussion should be conducted at a multidisciplinary tumor board when treating such patients with N0 neck.

Though the univariate analysis conducted herein did not show any significant clinicopathological predictors for cRF, some cRF predictors have been previously reported (Table 3). Vergeer et al. showed that the number of nodes involved in the ipsilateral neck is a prognostic factor for cRF [5]. Hence, a higher N stage might impact the cRF rate. Liu et al. reported that a tumor depth of invasion (DOI) of >10 mm is a significant predictor in small (T1–2) lateralized OSCCs (HR: 6.7, 95% CI 1.4–32.3, *p* = 0.02) [11], which indicates that a higher T stage from patients diagnosed with early T1/2 in the AJCC 7th staging system can be revised to T3/4 in the DOI-incorporated edition of the AJCC 8th staging system. It might also impact the cRF rate. As our study mainly used the AJCC 6th and 7th staging systems, some T1-2N0 cases that were earlier excluded from our analysis might get upstaged if the AJCC 8th staging system is used. However, except for the influence of DOI, there were still no factors significantly associated with cRF in our study, possibly owing to the limited number of cRF cases.

Notably, contralateral neck recurrence was detected as the first site of failure in five patients in our cohort, all of whom also simultaneously exhibited local recurrence. No isolated contralateral neck recurrence was observed. The failure pattern between the local failure and the cRF of other studies was also reviewed in Table 3. Wirtz reported simultaneous local and contralateral regional failure in 42% of patients with cRF [12]. Contreras reported that half of cRF occurred simultaneously with local failure and the other half occurred following the local failure [15]. Though these studies did not conduct any statistical tests of the correlation between local failure and cRF, the results implied that contralateral neck should be more closely examined or followed up when local failure was found. It is generally considered that patients with a history of dissection or RT in the neck may have aberrant lymphatic drainage caused by the disruption of lymphatic channels. This concept was verified and tested by SLNB in a new study, given the result of unexpected drainage pattern variability in 30% of patients with cT1-2N0 OSCC [25]. Hence, SLNB has become an emerging technique that can benefit the staging of the contralateral negative neck and avoid the overtreatment of the contralateral neck in the future [26]. Achieving better local control might still be a priority in clinical situations for patients with a low risk of contralateral nodal recurrence.

The study has several limitations. First, the study could have a selection bias because of its retrospective nature; patients with more advanced stage or risks are likely to receive more intense treatments such as bilateral neck dissection or irradiation. Second, distance of the primary tumor to the midline was likely not measured with standardization. Third, the multivariable analysis should be considered exploratory because of the low number of cRF events, which limits its statistical power. Fourth, toxicity profiles were not assessed in this retrospective study. Although we could not compare the side effects between UNI to BNI in this study, we believed that eliminating coverage of one side of neck volume would significantly decrease the exposure dose to the neighboring normal organs and tissues, leading to fewer possible associated side effects. Furthermore, for a group of highly consistent subsites of OSCC, our results are only applicable to well-lateralized buccal, cheek, gum, and retromolar OSCC populations; therefore, extrapolating the results to ENE-positive, tumor cross-midline, or other head and neck cancers is not applicable. Nonetheless, this study shows a reliably low cRF rate in well-lateralized OSCC with locally advanced stage and provides support for the possibility of omitting treatment to the contralateral neck in this group. At present, we are designing a more rigorous study to prospectively validate these results.

## 5. Conclusions

The rate of cRF was acceptably low in patients with well-lateralized OSCC, even with advanced stage of initially uninvolved contralateral neck. Local and ipsilateral recurrence remained the main pattern of relapse, underlining the importance of RT to the primary and ipsilateral lymph nodes. Omitting contralateral neck irradiation with active surveillance could be safely considered without compromising the cure rate in patients with such locally advanced OSCC.

## Figures and Tables

**Table 1:** Characteristics of patients.

Covariate	Total	UNI Group	BNI Group	*p*-Value	No Adjuvant RT Group	*p*-Value
No. of cases	149	44	34		71	
Sex (%)						
Female	4 (2.7)	1 (2.3)	1 (2.9)	1.000	2 (2.8)	0.979
Male	145 (97.3)	43 (97.7)	33 (97.1)		69 (97.2)	
Diagnosed age (median [IQR])	52.0 [47.0, 59.0]	56.0 [47.8, 62.0]	50.0 [45.3, 55.8]	0.018	52.0 [47.0, 61.0]	0.075
Cancer site (%)						
Buccal + cheek	95 (63.8)	31 (70.5)	25 (73.5)	0.744	39 (54.9)	0.243
Retromolar	8 (5.4)	3 (6.8)	1 (2.9)		4 (5.6)	
Gum	46 (30.9)	10 (22.7)	8 (23.5)		28 (39.4)	
AJCC pT (%)						
1	10 (6.7)	3 (6.8)	3 (8.8)	0.645	4 (5.6)	0.336
2	21 (14.1)	6 (13.6)	7 (20.6)		8 (11.3)	
3	32 (21.5)	10 (22.7)	4 (11.8)		18 (25.4)	
4A	78 (52.3)	22 (50.0)	16 (47.1)		40 (56.3)	
4B	8 (5.4)	3 (6.8)	4 (11.8)		1 (1.4)	
AJCC pN (%)						
0	97 (65.1)	27 (61.4)	15 (44.1)	0.171	55 (77.5)	0.001
1	27 (18.1)	5 (11.4)	9 (26.5)		13 (18.3)	
2B	25 (16.8)	12 (27.3)	10 (29.4)		3 (4.2)	
AJCC pStage (%)						
3	46 (30.9)	9 (20.5)	10 (29.4)	0.425	27 (38.0)	0.079
4A	95 (63.8)	32 (72.7)	20 (58.8)		43 (60.6)	
4B	8 (5.4)	3 (6.8)	4 (11.8)		1 (1.4)	
ND side (%)						
Bilateral	18 (12.1)	5 (11.4)	7 (20.6)	0.534	6 (8.5)	0.247
Left	69 (46.3)	23 (52.3)	16 (47.1)		30 (42.3)	
Right	62 (41.6)	16 (36.4)	11 (32.4)		35 (49.3)	
Number of node examed (median [IQR])	27.0 [16.0, 35.0]	29.0 [23.8, 36.3]	32.5 [22.0, 44.8]	0.452	20.0 [13.5, 33.0]	0.001
Histology grade (%)						
1	74 (49.7)	18 (40.9)	21 (61.8)	0.090	35 (49.3)	0.323
2	68 (45.6)	23 (52.3)	13 (38.2)		32 (45.1)	
3	7 (4.7)	3 (6.8)	0 (0.0)		4 (5.6)	
Tumor size (median [IQR])	40.0 [27.0, 50.0]	40.0 [25.8, 50.0]	37.0 [30.0, 45.0]	0.840	40.0 [28.5, 50.0]	0.800
PNI (%)						
Negative	104 (69.8)	27 (61.4)	22 (64.7)	0.947	55 (77.5)	0.144
Positive	45 (30.2)	17 (38.6)	12 (35.3)		16 (22.5)	
LVI (%)						
Negative	121 (81.2)	37 (84.1)	23 (67.6)	0.150	61 (85.9)	0.068
Positive	28 (18.8)	7 (15.9)	11 (32.4)		10 (14.1)	
Surgical margin (%)						
Free	122 (81.9)	30 (68.2)	27 (79.4)	0.271	65 (91.5)	0.016
Close	13 (8.7)	8 (18.2)	2 (5.9)		3 (4.2)	
Positive	14 (9.4)	6 (13.6)	5 (14.7)		3 (4.2)	
Adjuvant treatment (%)						
Negative	71 (47.7)	NA	NA		71 (100.0)	
RT alone	42 (28.2)	24 (54.5)	18 (52.9)		NA	
CCRT	36 (24.2)	20 (45.5)	16 (47.1)		NA	
Concurrent CT regimen (%)						
Cisplatin	11 (30.6)	6 (30.0)	5 (31.3)	0.640	NA	
PF	19 (52.8)	11 (55.0)	8 (50.0)		NA	
PF+ Erbitux	1 (2.8)	0 (0.0)	1 (6.3)		NA	
Unknown	5 (13.9)	3 (15.0)	2 (12.5)		NA	
Smoking (%)						
Negative	24 (17.1)	7 (15.9)	3 (9.7)	0.662	14 (21.5)	0.342
Positive	116 (82.9)	37 (84.1)	28 (90.3)		51 (78.5)	
Betel nut chewing (%)						
Negative	31 (22.1)	8 (18.2)	4 (12.9)	0.770	19 (29.2)	0.147
Positive	109 (77.9)	36 (81.8)	27 (87.1)		46 (70.8)	
Alcohol drinking (%)						
Negative	52 (36.9)	18 (40.9)	8 (25.8)	0.268	26 (39.4)	0.347
Positive	59 (63.1)	26 (59.1)	23 (74.2)		40 (60.6)	

Abbreviations: IQR, interquartile range; UNI, unilateral neck irradiation; BNI, bilateral neck irradiation; AJCC, American joint committee on Cancer; ND, neck dissection; PNI, perineural invasion; LVI, lymphovascular invasion; RT, radiotherapy; CCRT, concurrent chemoradiation therapy; CT, chemotherapy; PF, cisplatin and fluorouracil; NA, not applicable; *p*, *p*-value.

**Table 2:** Univariate analysis of factors associated with cRF.

Univariate Analysis	Hazard Ratio (95% CI)	*p*-Value
pT Stage (T3–4 vs. T1–2)	0.367 (0.06–2.20)	0.272
pN number (positive vs. negative)	3.117 (0.52–18.67)	0.213
PNI (presence vs. absence)	1.753 (0.29–10.51)	0.539
LVI (presence vs. absence)	1.190 (0.13–10.65)	0.876
Margin (close vs. negative)	3.369 (0.35–32.40)	0.293
Margin (positive vs. negative)	3.212 (0.33–30.96)	0.313

Abbreviations: cRF, contralateral neck failure; PNI, perineural invasion; LVI, lymphovascular invasion; CI, confidence interval.

**Table 3:** Literature review of failure rates in the node-negative contralateral neck of oral cavity cancers.

Study	*n*	Primary Site	Lateralized Primary	ND	pT Status	pN Status	Adjuvant Tx	RT Neck Side	Rate of cRF	Predictors of cRF
Present study	149	BUC (63.8%) GUM (30.9%) RMT (5.4%)	Y	Uni (88%) Bil (12%)	T1 (6.7%) T2 (14.1%) T3 (21.5%) T4 (57.7%)	N+ (34.9%) N0 (65.1%)	NRT (47.7%) RT (28.2%) CRT (24.2%)	56.4% UNI 43.6% BNI	5 year rate 3.6% Crude rate 3.4%	None (100% SLRF)
Liu (2021) [11]	176	OT (82%) FOM (18%)	Y	Uni (100%)	T1 (68%) T2 (32%)	N+ (12%) N0 (81%) UNK (7%)	NRT (83%) RT (17%)	100% PTB ± UNI 0% BNI	2 year rate 3.6% 5 year rate 4.3% Crude rate 5%	DOI > 10 mm (22% SLRF; 22% LFTR; 56% iCLF)
Waldram (2020) [20]	101	OT (52%) FOM (17%) BUC (7%) GUM (15%) RMT (10%)	NR	Uni (69%) Bil (27%)	T1 (11%) T2 (40%) T3 (9%) T4 (40%)	N+ (63%) N0 (37%)	RT (75%) CRT (25%)	43% UNI 53% BNI	Crude rate 5%	NR
Contreras (2019) [15]	72	Oral cavity (20%) Oropharynx (51%) Hypopharynx (6%) Larynx (22%)	No, 71% involved/cross midline	Uni (8%) Bil (92%)	T1-T2 (49%) T3-T4 (51%)	N2-N3 (58%) N0-N1 (42%)	RT (53%) CRT (47%)	24% PTB 76% UNI 0% BNI	5 year contralateral neck control 94.5% Crude rate 2.8%	NR (50% SLRF; 50% LFTR)
Wirtz (2019) [12]	197	Oral cavity (38%) Oropharynx (53%) Hypopharynx (10%)	Y	Uni (27%) Bil (73%)	T0 (1%) T1 (36%) T2 (41%) T3 (10%) T4 (11%)	N+ (80.7%) N0 (19.3%)	RT (70%) CRT (30%)	100% UNI 0% BNI	5 year contralateral neck control 94.6% Crude rate 6.1%	None (42% SLRF)
O’steen (2019) [13]	32	OT (72%) FOM (28%)	Y	Uni (78%) Bil (22%)	T1 (47%) T2 (41%) T3 (9%) T4 (3%)	N+ (19%) N0 (81%)	RT (62%) CRT (38%)	41% PTB 59% UNI 0% BNI	Crude rate 0%	NR
Nobis (2017) [14]	150	OT (100%)	Y	Uni (70%) Bil (30%)	T1 (71%) T2 (29%)	N+ (23%) N0 (77%)	NRT (75%) RT (19%) CRT (6%)	NR	Crude rate 2.7%	NR
Vergeer (2010) [5]	123	Oral cavity (85%) -OT (25%) -FOM (8%) -BUC (19%) -GUM (48%) Oropharynx (15%)	No, 7% close/ cross midline	Uni (83%) Bil (0%)	T1 (22%) T2 (35%) T3 (11%) T4 (33%)	N+ (41%) N0 (59%)	RT (100%)	10% PTB 90% UNI 0% BNI	5 year contralateral neck control 92% Crude rate 5.7%	Number of positive nodes (14% SLRF)

Abbreviations: BUC, buccal; RMT, retromolar trigon; OT, oral tongue; FOM, floor of mouth; NR, not reported; ND, neck dissection; Uni, unilateral; Bil, bilateral; UNK, unknown; Tx, treatment; RT, radiotherapy; CRT, concurrent chemoradiation therapy; NRT, no radiotherapy; UNI, unilateral neck irradiation; BNI, bilateral neck irradiation; PTB, primary tumor bed; cRF, contralateral neck failure; SLRF, simultaneous local recurrence failure; LFTR, local failure then neck recurrence; iCLF, isolated contralateral failure; DOI, depth of invasion.

## Data Availability

The datasets used and/or analysed during the current study are available on reasonable request from the corresponding author.
